# The GUIDE-CDSS framework: a comprehensive framework to GUIDE and evaluate the implementation of Clinical Decision Support Systems in healthcare, based on an overview of reviews

**DOI:** 10.1093/jamia/ocag080

**Published:** 2026-06-12

**Authors:** Esther R C Janssen, Jesper Knoop, Gianmatteo Farabolini, Nicoló Baldini, Thomas Hoogeboom, Maria Gabriella Ceravolo, Stefano Negrini, Carlotte Kiekens, PREPARE Project Group, Aghayev Emin, Alepopoulos Vasilis, Banfi Giuseppe, Calcina Erik, Capecci Marianna, Maria Gabriella Ceravolo, Cordani Claudio, Ferrario Irene, Georgiadis Charalampos, Giannios Georgios, Gramatikopoulou Margarita, Herrmann Christian, Hoogendam Lisa, Thomas Hoogeboom, Esther Janssen, Khijmatgar Shahnawaz, Carlotte Kiekens, Jesper Knoop, Kompatsiaris Ioannis, Kotsis Vasilios, Mpaltadros Lampros, Alberto Negrini, Stefano Negrini, Nikolopoulos Spiros, Novak Erik, Patias George Robert, Patias Petros, Pennestrì Federico, Poli Arianna, Roustanis Themistocles, Selles Ruud, Staal Bart, Tsaopoulos Dimitrios, Tartaglia Gianluca Martino, Zaharatos Haris, Helena Burger, Zala Kuret, Gaj Vidmar, Zlatko Matjačić

**Affiliations:** Radboud Institute for Health Sciences, IQ Health, Radboud University Medical Centre, Nijmegen, 5600HB, The Netherlands; Musculoskeletal Rehabilitation Research Group, School of Allied Health, HAN University of Applied Sciences, Arnhem/Nijmegen, 6802 EE, The Netherlands; Department of Orthopedic Surgery, VieCuri Medical Centre, Venlo, 5912BL, The Netherlands; Musculoskeletal Rehabilitation Research Group, School of Allied Health, HAN University of Applied Sciences, Arnhem/Nijmegen, 6802 EE, The Netherlands; Department of Movement Sciences, Faculty of Behavioural and Movement Sciences and Amsterdam Movement Science Research Institute, Vrije Universiteit, Amsterdam, 1081 HV, The Netherlands; Department of Experimental and Clinical Medicine, Marche Polytechnic University, Ancona, 60121, Italy; Department of Experimental and Clinical Medicine, Marche Polytechnic University, Ancona, 60121, Italy; Department of Rehabilitation, Physiotherapy Sciences and Sport, Brain Center, University Medical Center Utrecht, Utrecht, 3508 GA, The Netherlands; Research Group Innovation of Human Movement Care, HU University of Applied Sciences Utrecht, Utrecht, 3508 AJ, The Netherlands; Department of Experimental and Clinical Medicine, Marche Polytechnic University, Ancona, 60121, Italy; Laboratory of Evidence-Based Rehabilitation, IRCCS Galeazzi—Sant’Ambrogio Hospital, Milan, 20157, Italy; Department of Biomedical, Surgical and Dental Sciences, University of Milan “La Statale”, Milan, 20122, Italy; Laboratory of Evidence-Based Rehabilitation, IRCCS Galeazzi—Sant’Ambrogio Hospital, Milan, 20157, Italy

**Keywords:** qualitative research, prediction algorithms, precision medicine, artificial intelligence, implementation science, decision support systems

## Abstract

**Objectives:**

Despite the significant potential for Clinical Decision Support Systems (CDSSs) to improve care processes and health outcomes, several barriers hinder their widespread implementation in healthcare. While numerous systematic reviews have summarized potential barriers and facilitators for CDSS implementation, a comprehensive framework to guide and evaluate the implementation of CDSSs in healthcare is lacking. This overview of reviews, aims to establish a framework—GUIDE-CDSS—aimed at guiding and evaluating implementation of CDSSs in healthcare.

**Materials and Methods:**

An overview of systematic and scoping reviews was conducted by searching 6 databases. Systematic reviews or scoping reviews that used qualitative research methods to described implementation determinants for CDSSs in the healthcare domain were included. The AMSTAR 2 tool was used to assess the methodological quality. Results were collated into the GUIDE-CDSS framework. This framework describes implementation determinants and elements within those determinants found to impact implementation of CDSSs in healthcare.

**Results:**

Twenty-three reviews were included in the analysis. All reviews had at least 2 critical weaknesses, showing a limited methodological quality of included reviews. Eight determinants and 38 elements for implementation of CDSSs in healthcare and were described in the GUIDE-CDSS framework: perceived relevance, perceived effect, trustworthiness, ease of use, workflow, training and skills, resources, and implementation strategy.

**Discussion and Conclusion:**

This overview provides a comprehensive synthesis of the determinants influencing the implementation of CDSSs in healthcare, collated in the GUIDE-CDSS framework. The findings underscore that for successful CDSS development, implementation and evaluation is multifactorial.

**Protocol registration:**

This study was registered in PROSPERO (No. CRD42024512455).

## Introduction

To maintain accessible, affordable, and high-quality healthcare, a transition is needed from a population-based, disease-specific approach to a person-centered care that can better address the diverse health needs of a growing global population.^1^ Person-centered care focuses on the ideal of whole-person care and shared decision making.^2^ This shift from a reliance on population-level evidence (ie, group averages) and generalized guidelines toward person centered care is ongoing and rapidly accelerating due to a number of factors: (1) the inherently person-centered nature of healthcare, which by definition requires a personalized approach,^3^ (2) the expanding range of diagnostic and treatment options,^4^^,^^5^ (3) the increasing demand for healthcare services amid limited resources,^6^ (4) the growing availability of large-scale, global datasets that remain largely untapped,^7^ (5) rapid development of evidence in healthcare.^8^ As a result Clinical Decision Support Systems (CDSSs) have emerged as critical tools for personalizing healthcare and enhancing clinical decision-making.^9^ These systems are increasingly used at the point of care, supporting individualized diagnostic, prognostic, and treatment decisions.^10^ CDSSs leverage advanced data-driven models to make patient-specific inferences based on population data. Early examples of CDSSs, such as the STarT Back Screening Tool^11^ and the OCTOPUS stratification tool,^12^ facilitate stratified healthcare by classifying patients into clinically meaningful subgroups. While these approaches have shown some benefit in improving health outcomes, the field is now shifting toward truly personalized healthcare.^3^ In this next phase, advanced, AI-driven CDSSs offer considerable promise. By applying machine learning techniques, AI-based systems can generate accurate, individualized insights, providing clinicians and patients with the information necessary to make well-informed treatment decisions.^13^ For example, a CDSS could predict the likely outcomes of various treatment options for a specific patient by analyzing data from similar cases, thereby guiding clinicians and patients toward the most effective treatment plan.

Despite the significant potential for CDSSs to improve care processes and health outcomes, several barriers hinder their widespread implementation. Foremost among these challenges is the limited availability of robust, validated prediction models,^14^ which is partly due to the absence of standardized methods for collecting critical data on factors influencing healthcare outcomes, such as personal characteristics (eg, resilience, health literacy, motivation) and socioeconomic variables.^7^ Although multiple frameworks address aspects of technology adoption and implementation, no single framework fully captures the range of determinants specific to CDSS implementation in healthcare, often necessitating the combined use or adaptation of existing models.^15–19^ This framework is necessary not only to plan for successful implementation but also to evaluate its effectiveness across diverse healthcare settings. Without such a framework, we cannot systematically identify and address the key determinants—such as organizational culture, technological infrastructure, and clinician engagement—that are crucial for enhancing the successful uptake of CDSSs.

The findings from this study will inform the development, implementation, and evaluation of an advanced Clinical Decision Support System (CDSS) within the EU Horizon-funded PREPARE project, showcasing the value of the proposed framework. PREPARE is a multinational project focused on creating data-driven tools for personalized, holistic rehabilitation and care. To support this aim, we conducted an overview of existing reviews to inform the development, implementation, and evaluation of the CDSS tools.

This overview of systematic and scoping reviews describes the current qualitative evidence on the implementation determinants of CDSSs in healthcare settings and collates them into the GUIDE-CDSS framework.

## Methods

An overview of systematic and scoping reviews was performed to summarize published literature related to the implementation of CDSSs in healthcare. This study was registered in PROSPERO (No. CRD42024512455) and reported according to the PRISMA checklist and preferred reporting items for overviews of reviews (PRIOR).^20^^,^^21^ In this overview of reviews, we analyzed the qualitative results of the registered PROSPERO protocol. This decision was driven by the large number of eligible reviews and by the distinct analytical requirements of qualitative versus quantitative outcomes The quantitative results will be reported elsewhere.

### Literature search

A systematic search of 6 databases was conducted [MEDLINE (through PubMed), Cochrane, Embase, IEEE Xplore, ACM Digital Library, Episthemonikos] from January 1, 2014 until December 5, 2023. The limited timeframe was chosen to only include state-of-the-art evidence. Reference lists of included reviews were hand-searched for other reviews. Key search terms were related to “clinical decision support” and “systematic review.” The used search string for each database can be found in Appendix S1.

### Study selection

Studies were included if they were systematic reviews, meta-analyses or scoping reviews that used individual patient data and some type of statistical model to support clinical decision making (eg, patient profiling, risk assessment, diagnostic support, disease progression modeling, personalized treatment recommendations, outcome prediction, therapy response monitoring, or patient monitoring) within the healthcare context. We excluded rule-based systems, such as alarm systems, computerized reminders, and guideline-based process support tools. While these systems are often categorized under the term CDSS, they primarily provide standardized protocol support rather than enabling data-driven personalization of care, which was the central focus of this overview. Furthermore, studies needed to at least assess qualitative implementation outcomes of implementation of CDSSs. Exclusion criteria were: (1) if the functions of the included CDSS were patient safety monitoring, administrative functions or process improvement (eg, alarm systems, computerized reminders, computerized guidelines, patient data reports, documentation templates, clinical workflow tools), (2) non-English articles, and/or (3) reviews of model-based cost evaluations. Non-English articles were excluded to ensure the overview reflects the state-of-the-art applicable to an international audience. Given that non-English publications often report findings specific to a particular country or region, they were deemed less relevant for the purposes of this review.

Two pairs of reviewers (N.B. and J.K., E.R.C.J. and G.F.) independently conducted the study selection. Each pair screened half of the found studies, using the Rayyan software.^22^ First, title and abstract were screened for eligibility. Second, the full texts of the eligible articles were retrieved and reviewed. Discrepancies in all phases were solved during a consensus meeting with all 4 reviewers, and when necessary, an expert reviewer (C.K.) resolved final conflicts. If no full text was available a maximum of 3 attempts was made to contact the corresponding author. If the full text could not be retrieved, the study was excluded.

### Risk of bias

Four individual reviewers (E.R.C.J., G.F., N.B., and J.K.) applied the AMSTAR 2 tool to assess the methodological quality of the included reviews.^23^ The AMSTAR 2 is not intended to generate an overall score, but rather to assist in the identification of high-quality systematic reviews. Through the appraisal of the 16 AMSTAR 2 items, critical and non-critical weaknesses were identified in the context of our overview. Risk of Bias (RoB) assessments of included reviews were assessed to identify methodological weaknesses across reviews. The reviewers then judged the overall confidence in the results of the included reviews, based on combined Risk of Bias assessments and AMSTAR 2 results were possible.^24^ Disagreement between the reviewers was resolved through a consensus meeting with an expert reviewer (C.K.).

### Data extraction

Four reviewers (N.B., G.F., J.K., and E.R.C.J.) independently extracted data using Castor (version 2025.4.0; Castor, Amsterdam, the Netherlands). N.B. and G.F. cross-checked each other’s extractions, as did J.K. and E.R.C.J. The following data on included reviews were extracted: author(s), year of review, aim/scope of review, in- and exclusion criteria, time frame of inclusions, method of review, number of included papers, types of papers included, risk of bias assessment, major findings, recommendations/critical appraisal (eg, level of evidence table/GRADE table). Several dimensions of individual CDSS design features were recorded: type of CDSS, patient population, clinical task addressed, geographical coverage. Barriers and facilitators of CDSS implementation were extracted and tabulated using Excel (version 2408, Microsoft, Redmond, United States) as free text, to reflect implementation determinants.

### Synthesis of results

Descriptive statistics were reported for study characteristics and CDSS characteristics. A narrative review was conducted for all implementation outcomes using the SWiM guideline.^25^ Both the ENTREQ and eMERGe guidelines were considered not applicable as they guide reviews of primary studies. Qualitative data were described as implementation determinants when applicable and synthesized for similar outcomes into overarching implementation determinants and associated elements to improve their coherence. This grouping was performed using a thematic analysis approach, in which we drew initial elements from barriers and facilitators with similarities in semantics and meaning, reported by the reviews (source data). Thereafter, we identified broader implementation determinants reflecting meaningful concepts for implementing a CDSS in practice. All authors reviewed and refined these implementation determinants, during consensus rounds. Determinants were established through discussion with the involved authors until consensus was reached. We did not analyze if there was overlap of primary studies between the included reviews, as this would not influence the outcome of this overview. If overlap between identified barriers and facilitators was found they were comprised into a single element (see Appendix S2).

## Results

### Characteristics of included reviews

**Figure 1.**
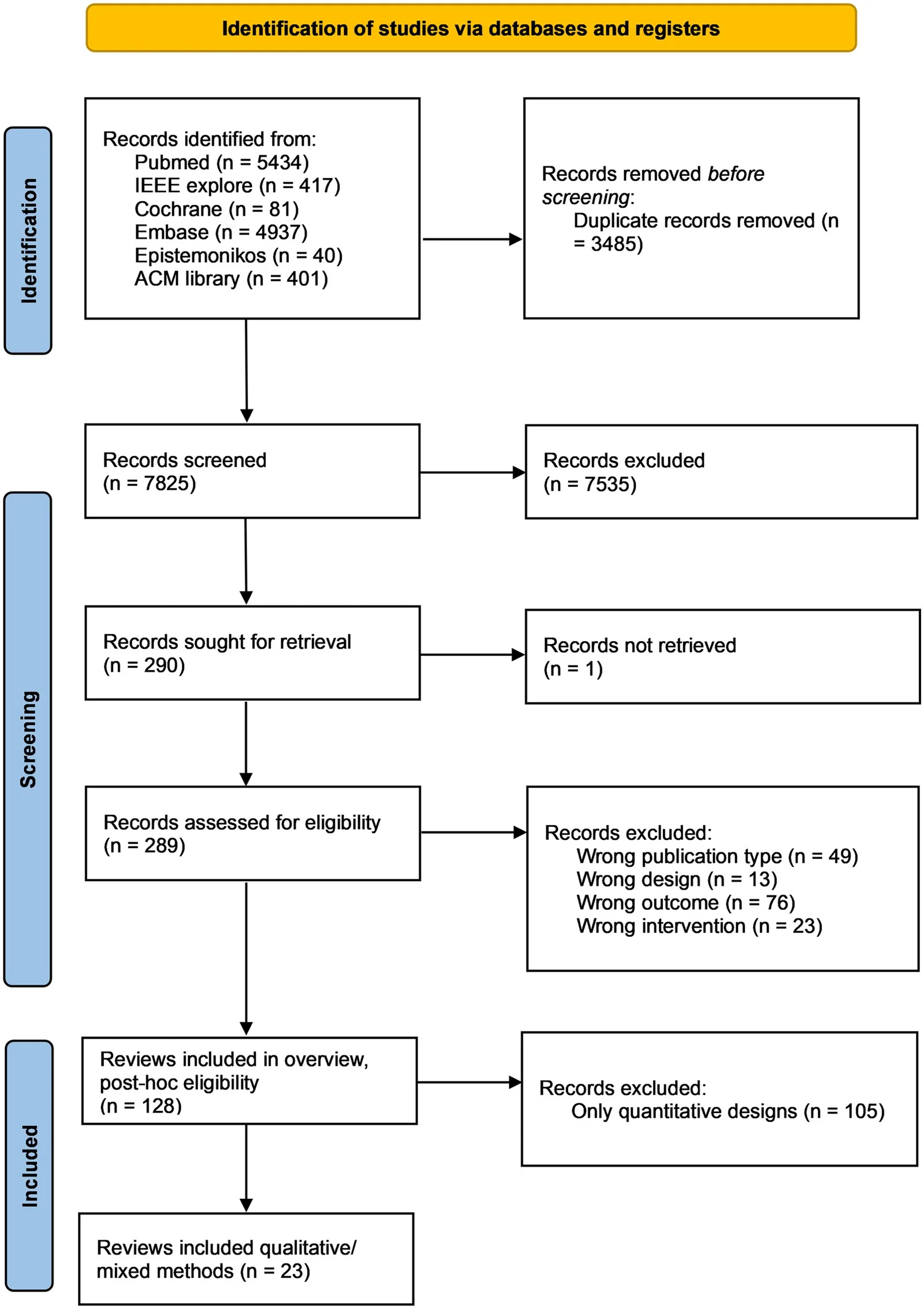
PRISMA flowchart.

Our search yielded 11 310 papers of which 13 reviews reporting mixed (both quantitative and qualitative) outcomes and 10 reviews reporting qualitative outcomes were eligible, see Figure  1. They included results from 615 primary studies from diverse medical fields. Included reviews reported on studies from Europe (*n* = 17), North America (*n* = 17), South America (*N* = 1), Africa (*n* = 4), Asia (*N* = 12) and Oceania (*N* = 11). We found 5 reviews from the field of general medicine, 3 from nursing, 3 from oncology, 2 from cardiology, 2 from genetics, 2 from infectious diseases, 1 from allied healthcare, 1 from emergency medicine, 1 from hospital care, 1 from internal medicine, 1 on COVID-19, and 1 with a generic hospital perspective. Details on the study characteristics can be found in Table S1. The authors described the use of CDSS in their respective fields in a qualitative manner.

### Assessment of quality

We applied the Amstar 2 to assess the methodological quality of the reviews, see Table S2. The domains on the appropriateness of meta-analysis, RoB on meta-analysis results and assessment of publication bias were not scored for any of the included reviews. As such 2 of the 7 critical domains were not assessed in this overview of reviews. This should be considered when interpreting the overall confidence in the results of our overview. There were no reviews without less than 2 critical weaknesses, there were 2 studies with 2 major weaknesses, 5 with 3 major weaknesses, 4 with 4 major weaknesses and 12 with 5 major weaknesses. Included reviews used varying tools, criteria, or thresholds to judge the RoB in primary studies, as such no synthesis of the risk of bias of primary studies or overall level of confidence assessment was performed.

### Implementation determinants

Our synthesis of the reviews identified 8 implementation determinants (ie, barriers and/or facilitators for implementation) from 38 elements. The elements within the implementation determinants describe the prerequisites for implementing CDSS in healthcare practice with more granularity. The implementation determinants and their associated elements are described in Table 1.

**Table 1.** Implementation determinants and elements

Implementation determinant	*N* of reviews	Elements	*N* of reviews
Perceived relevance	14	Relevance of the outcome of the CDSS	9^26–28^^,^^30–32^^,^^35–37^
		Relevance within the context	8^26–29^^,^^32^^,^^34^^,^^36^
		Personalization within the context	7^26^^,^^28^^,^^29^^,^^31^^,^^33^^,^^38^^,^^39^
		Timeliness of information	3^28^^,^^29^^,^^34^
		Alignment with organizational goals	2^26^^,^^28^
		Accuracy of the content	2^27^^,^^29^
Perceived effect	11	Influence on treatment outcome	9^26^^,^^29^^,^^30^^,^^32^^,^^33^^,^^36^^,^^38^^,^^40^^,^^42^
		Influence on communication	8^26^^,^^28^^,^^29^^,^^32^^,^^36^^,^^38^^,^^41^^,^^42^
		Influence on care process	8^26^^,^^29^^,^^30^^,^^32^^,^^33^^,^^38^^,^^40^^,^^43^
		Influence on patient and professional satisfaction	2^28^^,^^32^
Trustworthiness	16	Attitude toward CDSS	11^26^^,^^28^^,^^31–34^^,^^38^^,^^40^^,^^42–44^
		Respect for user autonomy	7^26^^,^^28^^,^^32^^,^^36^^,^^38^^,^^41^^,^^42^
		Organizational culture	7^26^^,^^28^^,^^29^^,^^32^^,^^33^^,^^39^^,^^44^
		Reliability of information	5^26^^,^^28^^,^^30^^,^^32^^,^^36^
		Medicolegal compliance	3^26^^,^^28^^,^^41^
		Performance of underlying model	1^28^
Ease of use	15	Integration within current systems	11^26–29^^,^^31^^,^^32^^,^^34^^,^^36^^,^^39^^,^^41^^,^^43^
		Available functionalities	11^26–29^^,^^31–33^^,^^36^^,^^38^^,^^44^^,^^45^
		CDSS fit for purpose	9^26–29^^,^^32^^,^^33^^,^^36^^,^^39^^,^^45^
		Technical compatibility	6^30^^,^^32–34^^,^^36^^,^^39^
		Look and feel of interface	6^29^^,^^32^^,^^36^^,^^39^^,^^44^^,^^45^
		Standardization of data	2^26^^,^^39^
Workflow	17	Integration within usual workflow	13^26–32^^,^^34^^,^^36^^,^^39^^,^^43^^,^^44^
		Time and effort to use CDSS	10^26–29^^,^^31–33^^,^^36^^,^^41^^,^^43^
		Appropriateness of alerts	8^26^^,^^27^^,^^29^^,^^31^^,^^32^^,^^35^^,^^39^^,^^41^
		Providing structure and guidance	4^28^^,^^29^^,^^32^^,^^41^
Training and skills	14	Training and education	12^26–33^^,^^38^^,^^41^^,^^44^^,^^45^
		Knowledge of CDSS	9^26^^,^^27^^,^^29–31^^,^^33^^,^^34^^,^^39^^,^^41^
		Supports knowledge	5^26^^,^^28^^,^^29^^,^^32^^,^^38^
		Technical skill level	4^26^^,^^32^^,^^33^
Resources	12	Financial	7^26^^,^^29^^,^^31^^,^^32^^,^^37–39^
		Technical	5^27^^,^^29^^,^^32–34^
		Staff capacity	5^26^^,^^28^^,^^29^^,^^31^^,^^41^
		Vendors	1^26^
Implementation strategy	9	Implementation planning	5^26^^,^^28^^,^^29^^,^^33^^,^^39^
		Incentives for use	5^28^^,^^31^^,^^32^^,^^34^^,^^36^
		Local champions	2^26^^,^^39^
		Advocated by professional/regulatory body	1^28^

1. Perceived relevance
  Perceived relevance refers to the degree to which the CDSS is perceived to align with the clinical context in which it is (intended to be) implemented.^26–39^ The 6 elements of perceived relevance were: relevance of the outcome of the CDSS,^26–29^^,^^32^^,^^34^^,^^36^ relevance within the context,^25–28^^,^^31^^,^^33^^,^^35^ personalization within the context,^24–27^^,^^30^^,^^32^^,^^34^ timeliness of information,^23–26^^,^^29^^,^^31^^,^^33^ alignment with organizational goals,^22–25^^,^^28^^,^^30^^,^^32^ and accuracy of the content.^21–24^^,^^27^^,^^29^^,^^31^
2. Perceived effect
  Perceived effect refers to the degree to which the CDSS is perceived to have an influence on the outcome or process of care and was described as an implementation determinant by 11 reviews.^26^^,^^29^^,^^30^^,^^32^^,^^33^^,^^36^^,^^38^^,^^40–42^ The 4 elements of perceived relevance were: influence on treatment outcome, influence on communication, influence on care process, and influence on patient and professional satisfaction.
3. Trustworthiness
  Trustworthiness refers to the qualities of the CDSS that make the user (dis)trust the tool they are using, which was reported as an implementation determinant by 16 reviews.^26^^,^^28–30^^,^^32–36^^,^^38–44^ The 6 elements of trustworthiness were: attitude toward CDSS, respect for user autonomy, organizational culture, reliability of information, medicolegal compliance, and performance of underlying model.
4. Ease of use
  The ease of use of the CDSS refers to the degree to which the system is being user-friendly in practice and was described as an implementation determinant by 15 reviews.^26–29^^,^^31–34^^,^^36^^,^^39^^,^^41^^,^^43–45^ The 6 elements of ease of use were: integration within current systems, available functionalities, CDSS fit for purpose, technical compatibility, look and feel of interface, and standardization of data.
5. Workflow
  Workflow refers to the degree to which the implementation of the CDSS interrupts or facilitates the normal care process and was described as an implementation determinant by 17 reviews.^26–36^^,^^38–41^^,^^43^^,^^44^ The 4 elements of workflow were: integration within usual workflow, time and effort to use CDSS, appropriateness of alerts, and providing structure and guidance.
6. Training and skills
  Training and skills refers to the ability, knowledge or training of the user required to use the CDSS and was reported as an implementation determinant by 14 reviews.^26–34^^,^^38^^,^^39^^,^^41^^,^^44^^,^^45^ The 4 elements of training and skills were: training and education, knowledge of CDSS, supports knowledge, and technical skill level.
7. Resources
  Resources refers to the availability of resources in the implementation setting to be able to implement the CDSS and was reported as an implementation determinant by 12 reviews.^26–29^^,^^31–34^^,^^37–39^^,^^41^ Elements of resources were: financial, technical, staff capacity, and vendors.
8. Implementation strategy

Implementation strategy refers to factors influencing the implementation process of a CDSS in healthcare and was described as an implementation determinant by 9 reviews.^26^^,^^28^^,^^29^^,^^31–34^^,^^36^^,^^39^ The 4 elements of implementation were: implementation planning, incentives for use, local champions, and advocated by professional/regulatory body.

### Other qualitative results

Three studies provided only narrative results on implementation of CDSSs, they reported their findings in a purely narrative format, without predefined categories or structured lists of determinants.^46–48^ Additional interpretative coding was used to identify and classify implementation determinants from these reviews prior to inclusion in the synthesis. They described implementation determinants already described in the synthesis: availability of data in clinical practice, generalizability of the CDSS, alert fatigue and integration of the CDSS with the information system. These can be allocated to the following elements: ease of use, perceived relevance and workflow

### GUIDE-CDSS

**Figure 2.**
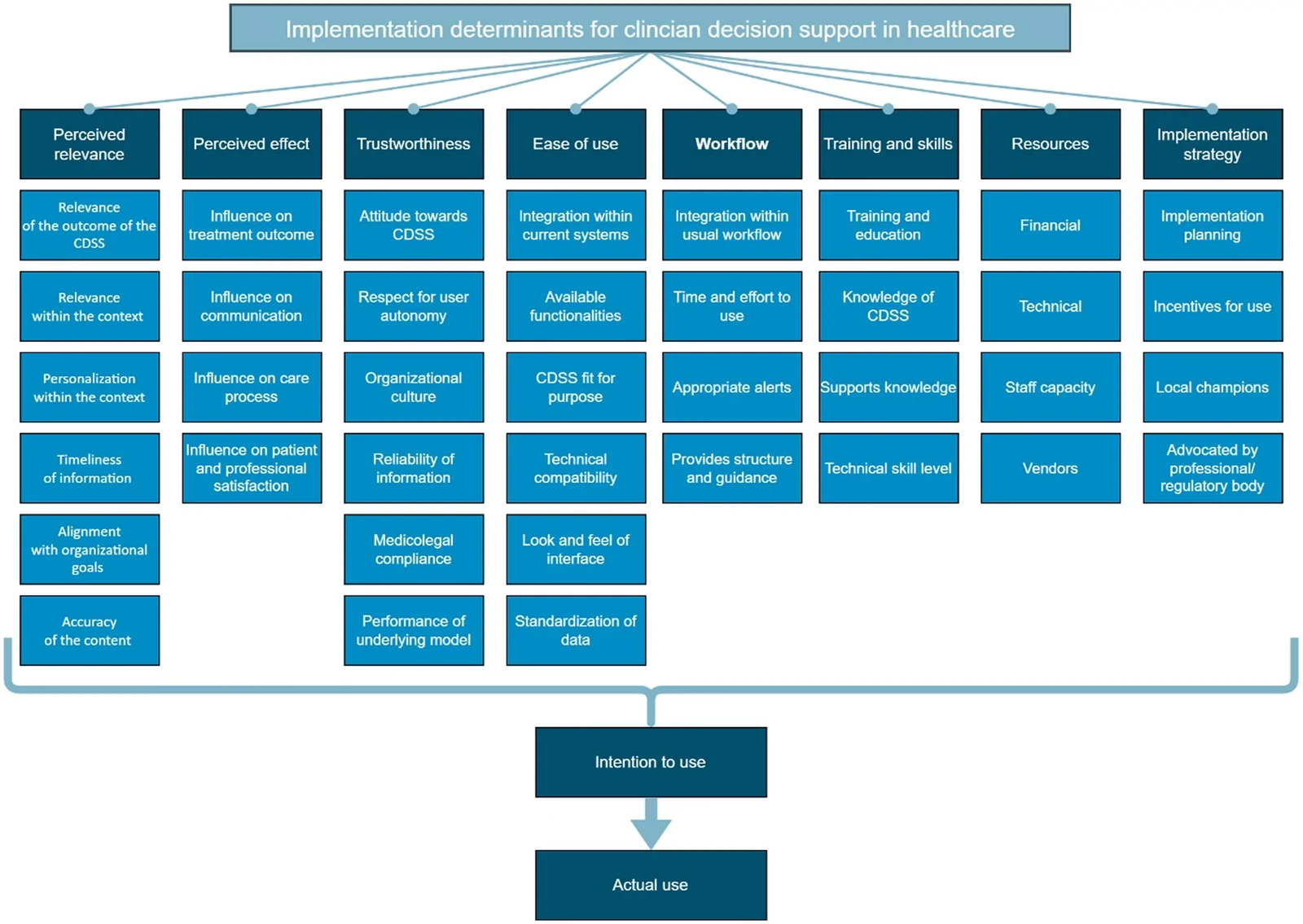
Framework of implementation determinants of clinical decision support systems in healthcare.

In Figure 2, we provide the GUIDE-CDSS framework: a comprehensive framework to guide and evaluate the implementation of clinical decision support systems in healthcare. The GUIDE-CDSS framework describes the implementation determinants that contain both barriers and facilitators for CDSS implementation in healthcare. This framework identifies the key challenges that must be addressed during the development phase of a CDSS making sure the tool aligns with all determinants for that specific context. During the implementation phase, these determinants should guide the process of integration into clinical practice to ensure successful adoption and functionality. Furthermore, the framework outlines measurable concepts that can be used to assess the implementation of CDSSs and identify potential factors contributing to unexpected outcomes after implementation.

## Discussion

The aim of this overview of reviews was to identify the perceived implementation determinants for implementing data-driven CDSSs in healthcare in general. Of the 7825 eligible reviews we included 23 reviews. The majority of these had critical weaknesses indicating an overall high risk of bias. We found a total of 8 concepts representing implementation determinants (ie, barriers and/or facilitators for implementation) of CDSSs in healthcare comprising 38 elements. These implementation determinants and their elements together from the GUIDE-CDSS framework for guiding and evaluating the implementation of CDSSs in healthcare.

In our overview of reviews we found that many of the reported determinants for implementing CDSSs are inverses of one another. For instance, while limited perceived relevance, lack of training, and insufficient trustworthiness act as barriers, their opposites—perceived relevance, adequate training, and sufficient trustworthiness—emerge as facilitators. Interestingly, many reviews reported either barriers or facilitators, but rarely both, potentially reflecting the focus or orientation of individual studies rather than the full spectrum of implementation dynamics. A more balanced approach that concurrently examines both sides of these constructs, as in this overview, provides a more comprehensive framework into how to optimize CDSS adoption.

The multifactorial challenges of CDSS implementation align closely with established theoretical models of technology adoption and implementation. We have made an overview that compares the implementation determinants of these widely applied frameworks in Appendix S3. Models such as the Unified Theory of Acceptance and Use of Technology (UTAUT),^16^ the Technology Acceptance Model (TAM),^17^ and the Technology Integration model (TIM)^18^ offer valuable perspectives on how users’ perceptions of usefulness, ease of use, and social or organizational influence shape adoption behaviors. These frameworks underscore the critical role of individual-level cognitive and motivational processes in determining acceptance of digital health innovations. However, while such models focus primarily on individual and technological determinants, the broader context of healthcare delivery often involves other factors, such as organizational variables, context of patient care or external factors (eg, policy documents, regulatory affairs), that are not fully captured in these technology acceptance theories.^19^ Moreover, these models are only partly applicable to the acceptance of technology in the medical field. To account for this, general implementation frameworks such as Proctor’s Implementation Framework provide complementary perspectives.^49^ This frameworks emphasize the importance of implementation strategies, fidelity, sustainability, and measurable implementation outcomes (eg, acceptability, feasibility, adoption, and penetration) to understand and assess embedding within an organization of care. The Consolidated Framework for Implementation Research (CFIR) provides a comprehensive overview of implementation determinants similar to our framework.^15^ Integrating insights from both technology acceptance and implementation science models can yield a more comprehensive understanding of successful CDSS implementation. Specifically, technology-focused models help explain why clinicians may or may not choose to use a support system, while implementation models help explain how and under what conditions these systems can be effectively integrated and sustained within complex healthcare environments. Schoville et al proposed the Integrated Technology Implementation Model (ITIM) in which both perspectives are combined^19^ and comprises 12 concepts that are central to the process of technology adoption. Many of these concepts fit our findings, although they miss the granularity described in our elements and therefore lack specific guidance for implementation of CDSSs in healthcare. Our framework bridges the gap between technology adoption models and implementation science frameworks, capturing both user-centered and contextual determinants within a single framework.

Despite the extensive theoretical understanding of technology implementation and adoption provided by these theoretical frameworks, the real-world adoption of CDSSs in the complex setting of healthcare remains limited.^50^ Particularly fields such as rehabilitation, due to the individualized person-centered care processes, could benefit from CDSSs, but these are poorly implemented.^51^ In such settings, it is paramount to consider all implementation determinants applicable to the specific context of CDSS implementation in healthcare described in this review to facilitate successful implementation.^52^ This means that a thorough implementation plan, taking into account all the implementation determinants in our framework, is crucial for the development of a CDSS as well as for its implementation into routine care. Within PREPARE-rehab we have used this framework to systematically identify relevant determinants during the development of our CDSS tools and planned pilot testing, supporting a more comprehensive implementation approach. For example, we have addressed perceived relevance issues by co-developing our tools with the intended end-users, so it will fit their clinical scope (eg, outcome selection) and communication process with the patient (eg, visualizations of predictions). Moreover, the implementation determinants from this framework can also be used as concepts for measurement instruments in the evaluation of implementation of CDSS in the healthcare setting. Importantly, rather than assuming equal relevance of all determinants across settings, the framework is intended to support the identification of context-specific priorities. For example, “appropriateness of alerts” may only be applicable for disruptive CDSSs. To operationalize this, we are translating the framework into a questionnaire. This instrument will enable healthcare organizations to identify and prioritize key barriers and facilitators, and to tailor implementation strategies accordingly.

We believe addressing these complex and interrelated challenges requires not only organizational and behavioral change, but as a prerequisite for tackling these more context-related challenges, we need to enable interoperability and facilitate trustworthy data analysis.^53^ In this regard, ongoing international efforts toward data standardization—such as the European Health Data Space (EHDS), and global collaborative initiatives like OHDSI (Observational Health Data Sciences and Informatics)—represent crucial enablers for the effective implementation of CDSSs and other digital health technologies.^54^ Although the OMOP Common Data Model is often associated with secondary use of data, its standardized vocabularies and data structures provide a semantic foundation that enables consistent interpretation of clinical data across systems, which is essential for scalable and interoperable CDSS logic. Emerging architectures that combine OMOP-standardized data with real-time interoperability standards such as FHIR demonstrate how harmonized data models can support point-of-care applications, effectively bridging the gap between retrospective analysis and real-time clinical decision support.^55^^,^^56^ These initiatives aim to harmonize data structures, terminologies, and governance mechanisms across healthcare systems, thereby reducing one of the most persistent technical barriers: the fragmentation and inaccessibility of high-quality clinical data across systems.^7^ By facilitating FAIR data (Findable, Accessible, Interoperable and Reusable) and secure data sharing, these frameworks create the preconditions necessary for higher-level adoption processes to take place.^57^ When data infrastructure is FAIR, healthcare organizations are better positioned to integrate decision support tools into clinical workflows, and clinicians are more likely to trust and engage with these systems.^58^ Thus, such data initiatives not only address the technological prerequisites for CDSS implementation but also act as catalysts for overcoming organizational and individual-level barriers.^59^^,^^60^

### Strengths and limitations

A key strength of this overview of reviews lies in its comprehensive scope and systematic synthesis of existing evidence on determinants for the implementation of CDSSs across diverse healthcare settings. By collating findings from a wide range of reviews, the study provides a broad understanding of the multidimensional factors influencing implementation, capturing both common and context-specific determinants. However, several limitations should be acknowledged. The inclusion of a large number of reviews necessitated the introduction of a post-hoc exclusion criterion to distinguish between reviews focusing on qualitative versus quantitative outcomes. While this aimed to improve analytical clarity, it may have introduced selective reporting and limited the integration of qualitative and quantitative evidence within the synthesis. Consequently, some of the richness and complementarity between different types of data may not have been fully captured. In addition, a RoB assessment of the primary studies was not performed due to the substantial heterogeneity in methods used for risk assessment across the included reviews.

## Conclusion

This overview of reviews provides a comprehensive synthesis of the implementation determinants influencing the implementation of CDSSs across healthcare settings. By identifying 8 overarching implementation determinants and their elements, the review highlights how technological, human, and organizational factors are interconnected. The findings underscore that successful CDSS implementation requires consideration of all implementation determinants, already during the development, but more importantly during implementation and evaluation of the CDSSs in healthcare, to increase their impact.

## Supplementary Material

ocag080_Supplementary_Data

## Data Availability

The data underlying this article are available in the article and in its online supplementary material.
