# Supplementary material for: The GUIDE-CDSS framework: a comprehensive framework to GUIDE and evaluate the implementation of Clinical Decision Support Systems in healthcare, based on an overview of reviews
Source: J Am Med Inform Assoc. 2026 Jun 12;33(9):1811–21. doi: 10.1093/jamia/ocag080 (PMC13580735; doi:10.1093/jamia/ocag080)
Supplement: Supplementary file 1 [file ocag080_supplementary_data.zip › Supplementary table 1. Study Characteristics..docx]

Supplementary table 1. Study Characteristics.

| **Study** | **Field** | **N included studies** | **Population** | **Intervention (CDSS definition)** | **Primary outcome** | **Included designs** | **Type of review** | **Study setting** | **Risk of bias tool** |
| --- | --- | --- | --- | --- | --- | --- | --- | --- | --- |
| Abell et al. 2023 | Hospital care | 44 |  | All decision support typologies, including both  knowledge-based and non-knowledge-based systems | Identify and map  key determinants of CDSS implementation and identify knowledge gaps. | Both qualitative and quantitative | Scoping review | Hospital | None |
| Akbar et al. 2021 | Nursing | 28 | Nurses | Not defined (electronic DSSs used solely by nurses) | Analyse effects of automated information processing on decision making, care delivery, and patient outcomes. | Both qualitative and quantitative | Systematic review |  | Joanna Briggs Institute’s |
| Borum et al. 2021 | Nursing | 9 | Nurse practitioners | Not defined | Not reported | Both qualitative and quantitative | Comprehensive literature review | Hospital | None |
| Bradley et al. 2021 | General medicine | 6 | GPs and oncology patients | CCDTs were defined as any tool used within a consultation which provided an outcome measure such as a percentage risk or a recommendation to the doctor to consider an underlying cancer  diagnosis as the cause for a patient’s signs or symptoms. | GPs’ attitudes to, and experiences of CCDTs. | Both qualitative and quantitative | Systematic search | Primary care | Critical Appraisal Skills Programme checklist |
| Burns et al. 2021 | General medicine | 26 |  | eRATs are computer-based algorithms designed to help clinicians avoid missing  important diagnoses, pick up possible symptoms early and  facilitate shared decision-making. | Examining how eRATs affect the communication and understanding of the risk of a particular disease, and uncertainty when used making diagnoses. | Qualitative studies | Systematic search | Primary care | Critical Appraisal Skills Programme |
| Chan et al. 2023 | Emergency medicine | 31 | Any relevant stakeholder | Any prediction model/score/algorithm containing at least 1 predictor and a numeric output. | At least 1 of the elements in the revised RE-AIM/PRISM framework  reported as a study outcome. This can be quantitative or qualitative. | Both qualitative and quantitative | Scoping review | Emergency room department | None |
| Chen et al. 2022 | Chronic diseases | 33 | Chronic diseases/healthcare providers | Contemporary EHR-based CDS system. | Describe healthcare provider experiences of implementing, using, evaluating, and sustaining EHR-based CDS interventions for chronic disease care. | Qualitative studies | Qualitative systematic review | Chronic disease care | JBI Checklist for Qualitative Research |
| van Dort et al. 2022 | Infectious diseases | 15 | Patients with antimicrobial resistance |  | Factors influencing acceptance. | Qualitative studies | Meta-synthesis | hospital | Joanna Briggs Institute Checklist for Qualitative Research |
| Fletcher et al. 2023 | General medicine | 91 | Healthcare professionals | Clinical decision support tools are used by health professionals to assist with clinical decision  making in relation to screening, diagnosis and management of a range of health conditions. | What is known from the existing literature about the use of eCDS tools by health professionals in general practice/primary care and the associated impact on workload and patient ‘flow’ through consulting sessions. | Both qualitative and quantitative | Systematic scoping review | General practice/primary care | none |
| Johnson et al. 2024 | Genetics | 41 |  | Computers to guide clinical decision-making and delivering genetically guided, patient-specific assessments and/or recommendations to healthcare providers and/or patients. | Barriers and facilitators of CDS implementation. | Both qualitative and quantitative | Systematic review | Genetics | none |
| Lee et al. 2020 | Generic | 44 |  | A model predicting a clinical outcome, use of EHR data for  modelling, automated data extraction for modelling, integration of the model into the EHR system, and  implementation into clinical use. our definition of “model” did require that there be some sort of  mathematical calculation involving predictors based on EHR data. | Review published peer-reviewed articles describing predictive models that have been implemented in real-world clinical practice, summarize their findings, and highlight lessons learned to further inform the literature regarding implementation of these emerging technologies in health care settings. | Qualitative studies | Systematic review | Clinical practice | Downs and Black checklist |
| Maes-Carballo et al. 2023 | Oncology | 17 | Patients with locally advanced breast cancer |  | Evaluated the existing DAs related to patients with LABC, their characteristics and usefulness, and identified possible improvement areas. | Both qualitative and quantitative | Systematic review |  | Qualsyst scoring system |
| Magalhaes Araujo et al. 2020 | Nursing | 16 | Nurses | Two types were considered in the classification of the clinical decision support systems: knowledge-based and non–knowledge based. Another classification used in this review divided the clinical decision support systems into 5 groups, according to their methodologies: machine learning, knowledge representation, information visualization, text mining, and multipurpose. | To identify the effects  of nurses using clinical decision support systems on clinical decision making for pressure ulcer management. Evaluation of these effects can clarify whether the incorporation of these systems in the workflow improves clinical nursing practice and  nurses' knowledge. | Both qualitative and quantitative | Systematic review | Pressure ulcer management | Mixed Methods Appraisal Tool |
| Mazo et al. 2020 | Oncology | 17 |  |  | What CDSSs are being used, how are they being  used, what are the advantages and disadvantages of CDSS use, and what are the effects of CDSS use  on patients with breast cancer. | Qualitative studies | Systematic review | Breast cancer | a questionnaire was completed for each paper |
| Meunier et al. 2023 | General medicine | 48 | Primary care  professionals | Clinical decision support systems (CDSSs) are software designed to be a direct aid to clinical decision making, in which an inference engine matches the features of an individual patient to a computerized clinical knowledge base or a machine learning algorithm and then presents patient-specific assessments or recommendations to the clinician or the patient for a decision. | Barriers and facilitators to the use of CDSSs by primary care professionals. | Both qualitative and quantitative | Systematic review |  | QuADS tool |
| Peters et al. 2023 | Cardiology | 5 |  | Digital DAs paper-based DAs with digital aspects. | Barriers and facilitators for the implementation of digital DAs for cardiovascular diseases. | Qualitative studies | Systematic review | Cardiovascular diseases | MMAT, CASP or Quality |
| Rittmann et al. 2019 | Infectious diseases | 45 | Physicians, mid-level  providers, nursing, and pharmacy | We defined CDSS as any electronic guidance tailored towards  direct antimicrobial and/or infection-related patient care recommendations to providers. This included automatic alerts  based on specific antibiotic use and patient symptoms,  algorithm-based questionnaires within guides, and specific  diagnoses. | Primary endpoints included time to antibiotic administration, mortality, adherence to recommendations/guidelines, antibiotic usage, CDSS usage, consultation usage, cost, coverage of organism, dosing, IV to PO, length of stay, urinalysis ordering rate, patient outcomes, user satisfaction, antibiotic resistance rate, performance metrics of developmental algorithms, total interventions made, and workflow metrics of developmental algorithms, total interventions made, and workflow. | Both qualitative and quantitative | Systematic review | Antibiotic stewardship in intensive  care unit, emergency departmen, and inpatient  care | Study quality was grossly estimated |
| Sebastian et al. 2020 | Genetics | 4 | Nongenetic clinicians within clinical practice | Genetic CDS tools were defined as tools providing assessments or recommendations based on the patient’s genotype, gene expression profile, or family history | The primary outcome was defined as  a change in management by the clinician. Secondary outcomes were changes in the risk assessment of patients’ disease by clinicians, changes in patient communication, clinician use of the CDS tool, and clinician satisfaction  with the CDS tool. | Both qualitative and quantitative | Systematic review |  | Joanna-Briggs Institute (JBI) quality assessment checklists |
| Skullbacka et al. 2022 | Cardiology | 21 | Any healthcare professional | The Risk Assessment Tool could involve anything from a risk score calculator  to using computer software or databases to assess the risk. | Summarize the evidence of different risk of assessment tools for QT prolonging pharmacotherapy. | Qualitative studies | Systematic review | any healthcare settings | Grading of Recommendations Assessment, Development, and Evaluation (GRADE) |
| Tebeje et al. 2021 | COVID-19 | 8 | SARS-COV-2 or COVID-19 |  | Describes how e-health can be used  to support the person-centered health care at the time of  COVID-19. | Both qualitative and quantitative | Systematic review |  | None |
| Toffaha et al. 2023 | Internal medicine | 39 |  | Leveraged an approach associated with AI and ML Al-algorithms for pressure injuries prediction and prevention | Any relevant outcome reported. | Qualitative studies | Comprehensive review | Hospital-acquired pressure injuries |  |
| Wosny et al. 2023 | Allied healthcare | 17 | Health Care Professionals |  | The benefits and challenges associated with the use of digital tools in hospital settings with a particular focus on the experiences of health care professionals using these tools. | Qualitative studies | Systematic review | Hospital | Critical Appraisal Skills Program |
| Yung et al. 2021 | Oncology | 10 | Only physicians treating patients with cancer  were included | Only computer-based decision tools used  to assist oncology consultations were included. Paper-based tools or digital tools such as websites that are used solely and independently by patients who seek information outside consultations with their treating physicians were not included. | Adoption of the technology for use during oncology consultations. | Qualitative studies | Systematic review | assisting shared decision-making during the  selection of anticancer therapy | Cochrane risk-of-bias tools |

Abbreviations: AI; artificial intelligence, CDSS; clinical decision support system, DA; decision aid, EHR; electronic health record, GP; general practitioner, LABC; locally advanced breast cancer, ML; machine learning, PO; oral ,IV; intravenous
