# Supplementary material for: The GUIDE-CDSS framework: a comprehensive framework to GUIDE and evaluate the implementation of Clinical Decision Support Systems in healthcare, based on an overview of reviews
Source: J Am Med Inform Assoc. 2026 Jun 12;33(9):1811–21. doi: 10.1093/jamia/ocag080 (PMC13580735; doi:10.1093/jamia/ocag080)
Supplement: Supplementary file 1 [file ocag080_supplementary_data.zip › Supplementary table 2. Amstar risk of bias assessment..docx]

Supplementary table 2. Amstar risk of bias assessment.

| AMSTAR 2 domain | Akbar et al. 2021 | Abell et al. 2023 | Borum et al. 2021 | Bradley et al. 2021 | Burns et al. 2021 | Chan et al. 2023 | Chen et al. 2022 | van Dort et al. 2022 | Fletcher et al. 2023 | Johnson et al. 2024 | Lee et al. 2020 | Maes-Carballo et al. 2023 |
| --- | --- | --- | --- | --- | --- | --- | --- | --- | --- | --- | --- | --- |
| PICO research question | Yes | Yes | Yes | Yes | Yes | Yes | Yes | No | Yes | No | No | Yes |
| Protocol registered* | Partial yes | Partial yes | No | No | Partial yes | No | Yes | No | No | No | Partial Yes | Partial Yes |
| Explanation of included designs | No | No | No | No | No | Yes | Yes | No | No | Yes | Yes | No |
| Adequacy of search* | Partial yes | Patial yes | No | Partial yes | Partial yes | No | Partial Yes | No | Partial yes | No | Partial yes | Partial yes |
| Duplicate study selection | Yes | Yes | No | Yes | Yes | Yes | Yes | Yes | Yes | Yes | Yes | Yes |
| Duplicate data extraction | No | Yes | No | No | No | Yes | No | No | No | No | yes | Yes |
| Justification of excluding studies* | Partial Yes | No | No | No | Partial Yes | No | Partial yes | No | No | No | Partial yes | Partial yes |
| Description of included studies | Partial Yes | Partial Yes | Partial Yes | Partial yes | Partial Yes | No | Partial yes | No | Partial yes | No | No | Yes |
| RoB review* | Yes | No | No | Yes | Partial Yes | No | Yes | Partial yes | No | No | Partial yes | Yes |
| Sources of funding reported | No | No | No | No | No | No | No | No | No | No | No | No |
| Appropriateness of meta-analysis* | - | - | - | - | - | - | - | - | - | - | - | - |
| RoB on meta-analysis results | - | - | - | - | - | - | - | - | - | - | - | - |
| Consideration of RoB in interpretation of results* | No | No | No | No | No | No | Yes | No | No | No | Yes | yes |
| Discussion of heterogeneity in results | Yes | No | No | No | No | No | No | No | No | No | No | Yes |
| Assessment of publication bias* | - | - | - | - | - | - | - | - | - | - | - | - |
| Disclosure of conflict of interest/funding | Yes | Yes | No | Yes | Yes | Yes | Yes | Yes | Yes | Yes | Yes | yes |
| N critical weakness | 4 | 5 | 5 | 4 | 5 | 5 | 2 | 5 | 5 | 5 | 4 | 3 |

| AMSTAR 2 domain | Magalhaes Araujo et al. 2020 | Mazo et al. 2020 | Meunier et al. 2023 | | Peters et al. 2023 | | | | Rittmann et al. 2019 | | Sebastian et al. 2020 | Skullbacka et al. 2022 | Tebeje et al. 2021 | | Toffaha et al. 2023 | Wosny et al. 2023 | | Yung et al. 2021 | |
| --- | --- | --- | --- | --- | --- | --- | --- | --- | --- | --- | --- | --- | --- | --- | --- | --- | --- | --- | --- |
| PICO research question | No | No | No | | Yes | | | | No | | Yes | No | No | | Yes | Yes | | Yes | |
| Protocol registered* | Partial yes | Partial yes | Partial yes | | Partial yes | | | | No | | Partial yes | Yes | No | | Partial yes | Partial yes | | Yes | |
| Explanation of included designs | No | No | No | | No | | | | No | | No | No | No | | No | Yes | | Yes | |
| Adequacy of search* | Partial yes | Partial yes | Partial yes | | Partial yes | | | | No | | Partial yes | Yes | Partial yes | | Yes | Partial yes | | Yes | |
| Duplicate study selection | Yes | No | Yes | | Yes | | | | No | | Yes | Yes | Yes | | No | Yes | | Yes | |
| Duplicate data extraction | yes | Yes | Yes | | No | | | | No | | Yes | Yes | No | | No | Yes | | No | |
| Justification of excluding studies* | Yes | No | No | | Partial yes | | | | No | | No | No | No | | Yes | Yes | | Partial yes | |
| Description of included studies | Yes | Partial yes | No | | Partial yes | | | | No | | No | Yes | No | | Yes | Partial yes | | Yes | |
| RoB review* | Yes | No | No | | Yes | | | | No | | Partial yes | Partial yes | No | | No | Yes | | No | |
| Sources of funding reported | No | No | No | | No | | | | No | | Yes | No | No | | No | No | | No | |
| Appropriateness of meta-analysis* | - | - | - | | - | | | | - | | - | - | - | | Yes | - | | - | |
| RoB on meta-analysis results | - | - | - | | - | | | | - | | - | - | - | | No | - | | - | |
| Consideration of RoB in interpretation of results* | Yes | No | No | | No | | | | No | | No | No | No | | No | No | | No | |
| Discussion of heterogeneity in results | No | No | No | No | | | | No | | No | | No | | No | No | | No | | No |
| Assessment of publication bias* | - | - | - | - | | | | - | | - | | - | | - | No | | - | | - |
| Disclosure of conflict of interest/funding | No | Yes | Yes | | | Yes | yes | | | Yes | | Yes | | Yes | Yes | | Yes | | Yes |
| Critical weaknesses | 2 | 5 | 5 | | | 4 | 5 | | | 5 | | 3 | | 5 | 3 | | 3 | | 3 |

* Critical AMSTAR 2 domains
