# Supplementary material for: The GUIDE-CDSS framework: a comprehensive framework to GUIDE and evaluate the implementation of Clinical Decision Support Systems in healthcare, based on an overview of reviews
Source: J Am Med Inform Assoc. 2026 Jun 12;33(9):1811–21. doi: 10.1093/jamia/ocag080 (PMC13580735; doi:10.1093/jamia/ocag080)
Supplement: Supplementary file 1 [file ocag080_supplementary_data.zip › Appendix 1. Literature search.docx]

Appendix 1. Literature search.

Search Strategy Medline

1# CDS terms:

"Decision Support Systems, Clinical"[Mesh] OR "Decision Support Systems, Management"[Mesh] OR "Decision Support Techniques"[Mesh] OR "Learning Health System"[Mesh] OR "decision support"[tiab] OR decision aid*[tiab] OR decision algorithm*[tiab] OR adaptive health system*[tiab] OR risk prediction*[tiab] OR clinical prediction rule*[tiab] OR clinical prediction tool*[tiab] OR clinical prediction model*[tiab] OR prognostic tool*[tiab] OR prognostic model*[tiab] OR adaptive health system [tiab]

2# Method terms:

("Meta-Analysis" [Publication Type] OR "Meta-Analysis as Topic"[Mesh] OR metaanaly*[tiab] OR meta-analy*[tiab] or metanaly*[tiab] OR "Systematic Review" [Publication Type] OR systematic[sb] OR "Cochrane Database Syst Rev"[Journal] OR prisma[tiab] OR preferred reporting items[tiab] OR prospero[tiab] OR ((systemati*[ti] OR scoping[ti] OR umbrella[ti] OR structured literature[ti]) AND (review*[ti] OR overview*[ti])) OR "Systematic Review" [Publication Type] OR systematic review*[tiab] OR scoping review*[tiab] OR umbrella review*[tiab] OR structured literature review*[tiab] OR systematic qualitative review*[tiab] OR systematic quantitative review*[tiab] OR systematic search and review[tiab] OR systematized review[tiab] OR systematised review[tiab] OR systemic review[tiab] OR systematic literature review*[tiab] OR systematic integrative literature review*[tiab] OR systematically review*[tiab] OR scoping literature review*[tiab] OR systematic critical review[tiab] OR systematic integrative review*[tiab] OR systematic evidence review[tiab] OR Systematic integrative literature review*[tiab] OR Systematic mixed studies review*[tiab] OR Systematized literature review*[tiab] OR Systematic overview*[tiab] OR Systematic narrative review*[tiab] OR ((systemati*[tiab] OR literature[tiab] OR database*[tiab] OR data-base*[tiab] OR structured[tiab] OR comprehensive*[tiab] OR systemic*[tiab]) AND search*[tiab]) OR (Literature[ti] AND review[ti] AND (database*[tiab] OR data-base*[tiab] OR search*[tiab])) OR ((data extraction[tiab] OR data source*[tiab]) AND study selection[tiab]) OR (search strategy[tiab] AND selection criteria[tiab]) OR (data source*[tiab] AND data synthesis[tiab]) OR ((critical[ti] OR rapid[ti]) AND (review*[ti] OR overview*[ti] OR synthes*[ti])) OR (((critical*[tiab] OR rapid*[tiab]) AND (review*[tiab] OR overview*[tiab] OR synthes*[tiab]) AND (search*[tiab] OR database*[tiab] OR data-base*[tiab]))) OR metasynthes*[tiab] OR meta-synthes*[tiab]) NOT ("Comment" [Publication Type] OR "Letter" [Publication Type] OR "Editorial" [Publication Type] OR (("Animals"[Mesh] OR "Models, Animal"[Mesh]) NOT "Humans"[Mesh]))

#1 AND #2 (> 2013)

Search Strategy Cochrane

1# CDS terms:

[mh "Decision Support Systems, Clinical"] OR [mh "Decision Support Systems, Management"] OR [mh "Decision Support Techniques"] OR [mh c] OR [mh "Decision Support Techniques"] OR "decision support":ti,ab OR ("decision" NEXT aid*):ti,ab OR ("decision" NEXT algorithm*):ti,ab OR ("decision support" NEXT tool*):ti,ab OR "clinical decision support system":ti,ab OR "clinical decision support systems":ti,ab OR ("clinical decision" NEXT support*):ti,ab OR ("medical decision" NEXT support*OR NEXT "adaptive health" NEXT system*):ti,ab OR ("risk" NEXT prediction*):ti,ab OR ("clinical prediction" NEXT rule*):ti,ab OR ("clinical prediction" NEXT tool*):ti,ab OR ("clinical prediction" NEXT model*):ti,ab OR ("prognostic" NEXT tool*):ti,ab OR ("prognostic" NEXT model*):ti,ab OR ("electronic clinical decision support" NEXT tool*):ti,ab OR ("computerized clinical decision support" NEXT system*):ti,ab OR "adaptive health system":ti,ab

2# Method terms:

(Meta-Analysis:pt OR [mh "Meta-Analysis as Topic"] OR metaanaly*:ti,ab OR meta-analy*:ti,ab OR metanaly*:ti,ab OR "Systematic Review":pt OR systematic OR "Cochrane Database Syst Rev":so OR prisma:ti,ab OR "preferred reporting items":ti,ab OR ((systemati*:ti OR scoping:ti OR umbrella:ti OR "structured literature":ti) AND (review*:ti OR overview*:ti)) OR "Systematic Review":pt OR ("systematic" NEXT review*):ti,ab OR ("scoping" NEXT review*):ti,ab OR ("umbrella" NEXT review*):ti,ab OR ("structured literature" NEXT review*):ti,ab OR ("systematic qualitative" NEXT review*):ti,ab OR ("systematic quantitative" NEXT review*):ti,ab OR "systematic search" AND review:ti,ab OR "systematized review":ti,ab OR "systematised review":ti,ab OR "systemic review":ti,ab OR ("systematic literature" NEXT review*):ti,ab OR ("systematic integrative literature" NEXT review*):ti,ab OR ("systematically" NEXT review*):ti,ab OR ("scoping literature" NEXT review*):ti,ab OR "systematic critical review":ti,ab OR ("systematic integrative" NEXT review*):ti,ab OR "systematic evidence review":ti,ab OR ("Systematic integrative literature" NEXT review*):ti,ab OR ("Systematic mixed studies" NEXT review*):ti,ab OR ("Systematized literature" NEXT review*):ti,ab OR ("Systematic" NEXT overview*):ti,ab OR ("Systematic narrative" NEXT review*):ti,ab OR ((systemati*:ti,ab OR literature:ti,ab OR database*:ti,ab OR data-base*:ti,ab OR structured:ti,ab OR comprehensive*:ti,ab OR systemic*:ti,ab) AND search*:ti,ab) OR (Literature:ti AND review:ti AND (database*:ti,ab OR data-base*:ti,ab OR search*:ti,ab)) OR (("data extraction":ti,ab OR ("data" NEXT source*):ti,ab) AND "study selection":ti,ab) OR ("search strategy":ti,ab AND "selection criteria":ti,ab) OR (("data" NEXT source*):ti,ab AND "data synthesis":ti,ab) OR ((critical:ti OR rapid:ti) AND (review*:ti OR overview*:ti OR synthes*:ti)) OR (((critical*:ti,ab OR rapid*:ti,ab) AND (review*:ti,ab OR overview*:ti,ab OR synthes*:ti,ab) AND (search*:ti,ab OR database*:ti,ab OR data-base*:ti,ab))) OR metasynthes*:ti,ab OR meta-synthes*:ti,ab) NOT (Comment:pt OR Letter:pt OR Editorial:pt OR (([mh Animals] OR [mh "Models, Animal"]) NOT [mh Humans]))

#1 AND #2 (> 2013)

Search Strategy Embase

1# CDS terms:

clinical decision support system/ or exp "decision support techniques"/ or learning health system/ or "decision support".ti,ab,kw,kf. or decision aid*.ti,ab,kw,kf. or decision algorithm*.ti,ab,kw,kf. or decision support tool*.ti,ab,kw,kf. or "clinical decision support system".ti,ab,kw,kf. or "clinical decision support systems".ti,ab,kw,kf. or clinical decision support*.ti,ab,kw,kf. or medical decision support*.ti,ab,kw,kf.or adaptive health system*.ti,ab,kw,kf. or risk prediction*.ti,ab,kw,kf. or clinical prediction rule*.ti,ab,kw,kf. or clinical prediction tool*.ti,ab,kw,kf. or clinical prediction model*.ti,ab,kw,kf. or prognostic tool*.ti,ab,kw,kf. or prognostic model*.ti,ab,kw,kf. or electronic clinical decision support tool*.ti,ab,kw,kf. or computerized clinical decision support system*.ti,ab,kw,kf. or adaptive health system.ti,ab,kw,kf.

2# Method terms:

(((exp meta analysis/ or exp "meta-analysis (topic)"/ or metaanaly*.ti,ab,kw,kf. or meta-analy*.ti,ab ,kw,kf. or metanaly*.ti,ab,kw,kf. or "systematic review"/ or "cochrane database of systematic reviews $".jn. or prisma.ti,ab,kw,kf. or preferred reporting items.ti,ab,kw,kf. or prospero.ti,ab ,kw,kf. or ((systemati* or scoping or umbrella or structured literature) and (review* or overview*)).ti. or systematic review*.ti,ab,kw,kf. or scoping review*.ti,ab,kw,kf. or umbrella review*.ti,ab,kw,kf. or structured literature review*.ti,ab,kw,kf. or systematic qualitative review*.ti,ab,kw,kf. or systematic quantitative review*.ti,ab,kw,kf. or systematic search.mp.) and review.ti,ab,kw,kf.) or systematized review.ti,ab,kw,kf. or systematised review.ti,ab,kw,kf. or systemic review.ti,ab,kw,kf. or systematic literature review*.ti,ab,kw,kf. or systematic integrative literature review*.ti,ab,kw,kf. or systematically review*.ti,ab,kw,kf. or scoping literature review*.ti,ab,kw,kf. or systematic critical review.ti,ab,kw,kf. or systematic integrative review*.ti,ab,kw,kf. or systematic evidence review.ti,ab,kw,kf. or systematic integrative literature review*.ti,ab,kw,kf. or systematic mixed studies review*.ti,ab,kw,kf. or systematized literature review*.ti,ab,kw,kf. or systematic overview*.ti,ab,kw,kf. or systematic narrative review*.ti,ab,kw,kf. or ((systemati* or literature or database* or data-base* or structured or comprehensive* or systemic*) and search*).ti,ab,kw,kf. or ((literature and review).ti. and (database* or data-base* or search*).ti,ab,kw,kf.) or ((data extraction or data source*) and study selection).ti,ab,kw,kf. or (search strategy and selection criteria).ti,ab,kw,kf. or (data source* and data synthesis).ti,ab,kw,kf. or ((critical or rapid) and (review* or overview* or synthes*)).ti. or ((critical* or rapid*) and (review* or overview* or synthes*) and (search* or database* or data-base*)).ti,ab,kw,kf. or metasynthes*.ti,ab,kw,kf. or meta-synthes*.ti,ab,kw,kf.) not (("letter" or "editorial").pt. or ((exp "animal"/ or exp "animal model"/) not exp "human"/)) [mp=ti, ab, hw, tn, ot, dm, mf, dv, kf, fx, dq, bt, nm, ox, px, rx, an, ui, sy, ux, mx]

#1 AND #2 (> 2013)

Search Strategy IEEE Xplore (limited to 9 wild cards)

1# CDS terms:

("All Metadata":"decision support") OR ("All Metadata":"decision aid") OR ("All Metadata":decision algorithm*) OR ("All Metadata":decision support tool*) OR ("All Metadata":"clinical decision support system") OR ("All Metadata":"clinical decision support systems") OR ("All Metadata":clinical decision support) OR ("All Metadata":medical decision support*) OR ("All Metadata":adaptive health system*) OR ("All Metadata":risk prediction*) OR ("All Metadata":clinical prediction*) OR ("All Metadata":prognostic tool) OR ("All Metadata":prognostic model*) OR ("All Metadata":electronic clinical decision support tool) OR ("All Metadata":electronic clinical decision support tools) OR ("All Metadata":"computerized clinical decision support system") OR ("All Metadata":"computerized clinical decision support systems")OR ("All Metadata": adaptive health system)

2# Method terms:

("Mesh_Terms":"Meta-Analysis as Topic") OR ("All Metadata":metaanaly*) OR ("All Metadata":meta-analy*) OR ("All Metadata":"Systematic Review") OR ("All Metadata":"Meta-Analysis") OR ("All Metadata":prisma) OR ("All Metadata":preferred reporting items) OR ("All Metadata":prospero) OR ("All Metadata":systematic review) OR ("All Metadata":scoping review) OR ("All Metadata":umbrella review) OR ("All Metadata":structured literature review) OR ("All Metadata":"systematic qualitative review") OR ("All Metadata":"systematic quantitative review") OR ("All Metadata":systematic search and review) OR ("All Metadata":systematized review) OR ("All Metadata":systematised review) OR ("All Metadata":systematic literature review) OR ("All Metadata":"systematic integrative literature review") OR ("All Metadata":"scoping literature review") OR ("All Metadata":"systematic critical review") OR ("All Metadata":"systematic integrative review") OR ("All Metadata":"systematic evidence review") OR ("All Metadata":"Systematic integrative literature review") OR ("All Metadata":"Systematic mixed studies review") OR ("All Metadata":"Systematized literature review") OR ("All Metadata":"Systematic overview") OR ("All Metadata":"Systematic narrative review") NOT (("All Metadata":"Comment") OR ("All Metadata":"Letter") OR ("All Metadata":"Editorial"))

#1 AND #2 (> 2013)

Search Strategy ACM Digital Library

1# CDS terms (abstract):

"Learning Health System" OR "decision support" OR decision aid* OR decision algorithm* OR decision support tool* OR "clinical decision support system" OR "clinical decision support systems" OR clinical decision support* OR medical decision support* OR adaptive health system* OR risk prediction* OR clinical prediction rule* OR clinical prediction tool* OR clinical prediction model* OR prognostic tool* OR prognostic model* OR electronic clinical decision support tool* OR computerized clinical decision support system* OR adaptive health system

2# Method terms:

Used review article filter

#1 + filer (> 2013)

Search Strategy Epistemonikos

1# CDS terms (abstract):

"Learning Health System" OR "decision support" OR decision aid* OR decision algorithm* OR decision support tool* OR "clinical decision support system" OR "clinical decision support systems" OR clinical decision support* OR medical decision support* OR adaptive health system* OR risk prediction* OR clinical prediction rule* OR clinical prediction tool* OR clinical prediction model* OR prognostic tool* OR prognostic model* OR electronic clinical decision support tool* OR computerized clinical decision support system* OR adaptive health system

2# Method terms:

Used review article filter

#1 + filer (> 2013)
