# Supplementary material for: The GUIDE-CDSS framework: a comprehensive framework to GUIDE and evaluate the implementation of Clinical Decision Support Systems in healthcare, based on an overview of reviews
Source: J Am Med Inform Assoc. 2026 Jun 12;33(9):1811–21. doi: 10.1093/jamia/ocag080 (PMC13580735; doi:10.1093/jamia/ocag080)
Supplement: Supplementary file 1 [file ocag080_supplementary_data.zip › Appendix 2.pdf]

Reported barriers (author arranged)

Determinant	Element	Abell et al. 20223	Akbar et al. 2021	Borum et al. 2018	Bradley et al. 2021	Burns et al., 2021	Chan et al, 2023	Chen et al. 2022	Fletcher et al, 2023	Johnson et al, 2024	Lee et al. 2020	Maes-Carballo et al. 2023	Araujo et al. 2020	Mazo et al 2020	Meunier et al, 2023	Peters et al.	Rittmann et al. 2019	Sebastian et al, 2020	Van Dort et al. 2022	Wosny et al., 2023	Yung et al. 2021	
Perceived relevance	Accuracy of content							Glitches and inaccuracies with alerts														
	Relevant within the context	Clarity of CDSS purpose		Incorrect format improperly defined/not current with evidence-based practice											professionals don't need help with the targeted health issue	Clinicians questioning if use of DA fosters SDM						
	Relevance of outcomes	Usefulness and relevance of CDSS and output in practice		Inaccurate patient information/lack of clinical patient information			Model outputs more relevant to staff than patients				guidelines could be easily memorized and thus did not require decision support				Recommendations are not helpful/relevant		the intervention was not perceived to be useful for the setting or the patient's	Concerns about clinical utility	providing outdated information		had already received on paper or during consultation / the technology did not provide all the information that patients reportedly found the language of computer-based decision tools too complex	
	Relevance of outcomes			Incorrect CDS content																		
	Information is provided timely						Model outputs delivered too late to be clinically useful									The timing of the DA does not fit into the care process and is challenging						
	Relevant within the context						Model not suited for the spectrum of disease in a different context	limited number of conditions addressed or patient factors							CDSS should target more health issues							
	Relevant within the context						(ability to impact clinical decisionmaking or enhance existing practice)															
	Personalized to context	CDSS not sensitive to complexity of patient or practice					The target condition is very rare	Guidelines applied indiscriminately to patients			"evolving clinical profiles"		Lack of flexibility									
Perceived effect	Influences treatment outcome		no significant effect on nursing interventions or cognitive load.																			
	Influences care process		documentation and plan of care compliance decreased after DSS implementation																			
	Influences care process		improved initially when DSS was implemented, it gradually deteriorated																			
	Influences treatment outcome		length of stay, which was increased or remained unchanged.																			
	Influences treatment outcome		patients with lower back pain also remained unchanged with the use of																			
	Influcence communication				Communicating, sharing and understanding risk			communication, priorities, and clinical relationship during the consult						over-emphasis on decision-making	Negative effect on patient-PCP communication							
	Influences treatment outcome																		negatively impacting workflow		some physicians perceived that the technology made patients somewhat more anxious.	
Workflow	Appropriate alerts	information overload/ alert fatigue		CDS alerts/nuisance alerts	Prompt fatigue			Too much information or too many alerts		alert fatigue	alert fatigue				alerts fatigue		"alert-fatigue" as an issue					
	Time and effort to use	time/effort to use or learn CDSS		CDS inefficiencies			Increased time and workload	Additional time required or timing was not right	Existing time/workload pressures		increased work burden on the care team		Lack of time for data input		with other PCPs to help physicians with CDSS's increased workload						the technology added 5 minutes to total consultation time	
	Time and effort to use								No time for preventive care		introduction of extra work discouraged use of several predictive models		Lack of time		Increased workload during the consultation							
	Integration into usual workflow			Inappropriate timing of CDS			model inputs or a completing tool because of practical circumstances	Disrupted and did not integrate with usual workflow	Workflow disruption - interruptive alerts/functions	clinical workflow disruptions	physician workflow by streamlining patient education and orders				using CDSS disrupts usual workflow	Implementing the DA in existing structures (education session)			being compatible with existing workflow and prescribing autonomy		Lack of incentives or infrastructure, time, information about treatment,	
	Integration into usual workflow								Change to the trajectory of conversation with patient						difficulty to use CDSSs for patients co-managed by other specialists							
Trustworthiness/accaptability	Reliability of information																					
	Reliability of information						model not validated or generalizable performance and/or certain clinical aspects not addressed by the model															
	Attitude towards CDSS					fear			Users feeling marked down by an external authority					CDSSs can be perceived as a threat to clinical judgment by some physicians,								
	Attitude towards CDSS													fear of learning or getting out of their comfort zone,								
	Attitude towards CDSS													fear of implementing new technology								
	Attitude towards CDSS		work environment, experience, and clinical judgment			mistrust	Lack of trust due to unfamiliarity (especially ML models)						Lack of trust and credibility in clinical decision support systems		questioning validity of CDSS's knowledge database	Concerns about the content of the DA				Unfavourable social structures		
	Attitude towards CDSS														doubtful reliability of the recommendations	Patients felt like clinicians selected parts of the DA and did not show it completely						
	Respect for user autonomy	CDSS challenges clinician autonomy/role			clinical acumen vs protocol		Perceived reduction in autonomy							physicians could think that CDSSs could limit their freedom to think	conflicts between CDSS recommendations and PCP expertise or beliefs						their own habits or preferences	
	Respect for user autonomy	use of CDSS compromises individual patient care																				
	Organizational culture	organisational governance/policy for CDSS						barriers to using and following CDS recommendations					Workplace culture									
	Medicolegal compliance	Medicolegal concerns			The medicolegal implications		Concerns about legal ramifications because of missed cases															
	Medicolegal compliance	security and privacy concerns of CDSS																				
Ease of use	Standardization of data	system level infrastructure to support CDSS								lack of standards for storing and transmitting genetic information												
	CDSS fit for purpose			Usability								format of presentation (barrier or facilitator)	Software design (also a facilitator)								technology difficult, disliked the use of tablet computers, and preferred written or printed material	
	CDSS fit for purpose												Lack of logical flow		the CDSS is not user-friendly format of recommendations (length, structure, font colors)						users needed to enter their username and password to log in	
	Integration with systems	technical dependencies and design of CDSS (also facilitator)		Lack of interoperability	Impact of IT integration		documentation of model scores (outside EMR or usual workflow)	Lack of integration and reliance on manual data collection	Slow software - being a separate system to EMR	lack of genetic data integration within the EHR	mapping the correct EHR fields for the desired data elements				CDSS not fully integrated in the EHR	prior to consultation, not integrated in electronic medical record					the technology was not readily available on the physicians' desktop.	
	Integration with systems	interoperability of CDSS with existing systems														Integrating the DA in the electronic health record					the technology was not integrated with the electronic health record.	
	Integration with systems																				integration with the electronic health record system,	
	Integration with systems																				availability of the technology on their desktops,	
	Available functionalities			Reliance on manual input							limitations in the predictive model's user functionality				need of customization options					Lack of a tailored tool design	physicians were not able to share information and treatment alternatives with their patients.	
	Available functionalities																			Insufficient design of user interface		
	CDSS fit for purpose											"overwhelming and difficult to process"			information overload							
	Technical compatibility												Frustration with clinical decision support systems use		CDSS slowness	Technical difficulties for playing the video			difficulties with access, and the complexity and time involved in completing tasks		encountered technical issues every time they tried to use the technology	had difficulty connecting wirelessly to the internet-
Implementation strategy	Implementation planning	implementation planning and strategies for CDSS								lack of patient engagement materials			Lack of information about the clinical decision support systems implementation									
	Incentives						Results not shared				providing incentives and reminders to encourage use of the model				lack of engagement from professionals (inertia of previous practice)	complete the decision aid as they felt anxious for the upcoming angiography					physicians did not take advantage of the information conveyed through the technology.	

	**Incentives**																	Half of the patients declined to use the tablet device and preferred to complete the study on paper
**Training and skills**	**Technical skill level**	Lack of technical skills/support for CDSS								Lack of computer skills		lack of computer skills						
	**Knowledge of CDSS**	clinician knowledge of CDSS, condition or data inputs	Lack of knowledge				lack of understanding of genetic information by the clinician	capability, opportunity, and motivation		Resistance to use computers			Lack of understanding of the concept of SDM 27 or on how to use the DA			knowledge in us- ing or understanding the intervention.		
	**Knowledge of CDSS**	Awareness of CDSS			Lack of visibility of interventions outside the ED	Not aware or does not see a need for tool							Little communication among team members about the DA	tool's visibility		ack of awareness of the intervention		
	**Knowledge of CDSS**													know that it existed and used the traditional nomogram		Lack of trust or familiarity with technology		
	**Traning and education**		Lack of training	One size fits all approach to training	education (especially for complex models like neural networks)			lack of training or lack of familiarity by rotating trainees	low educational level	Lack of training		training before use is needed				lack of proficient training.	Lack of training	
	**Traning and education**							educating providers about the model										
**Resources**	**Financial**	uncertain funding for CDSS				financial limiatations	with inadequate information or analysis on cost-benefit	requiring access to costly data			initial cost—e.g., maintenance, support, and training required	ask for financial compensation to use the CDSS			Concerns about costs for patients			
	**Vendors**	vendor considerations																
	**Technical**		Hardware issues							Lack of computer infrastructure		lack of computers or tablets	Rooms for use of one screen together with patient					
	**Staff capacity**	organisational capacity/constraints for use of CDSS		Time as a resource	Insufficient resources to deal with consequences of model implementation	resource limitations		overconsumption of resources,										

Reported facilitators (author arranged)

Determinant	Element	Abell et al. 20223	Akbar et al. 2021	Bradley et al. 2021	Burns et al., 2021	Chan et al, 2023	Chen et al. 2022	Fletcher et al, 2023	Johnson et al, 2024	Lee et al. 2020	Maes-Carballo et al. 2023	Magalhaes Araujo et al. 2020	Mazo et al 2020	Meunier et al, 2023	Peters et al.	Van Dort et al. 2022	Wosny et al., 2023	Yung et al. 2021
Ease of use	Integration with systems	CDSS integration and fit with workflows				Existing infrastructure and/or processes (eg. EMR, CDS)		tools that were embedded in the EMR	genetic data integration within EHR						Difficult access to the tool prior to consultation, not integrated in electronic			
	Integration with systems					Existing systems and processes for screening target condition									Integrating the DA in the electronic health record			
	Integration with systems					Incorporation into routine workflow documentation												
	CDSS fit for purpose	Practical usability of CDSS				Ease of use, well organized, interface user friendly and interactive	System is easy to use and reliable		guideline accessibility			Easy to use		CDSS recommendations are easy to understand				
	CDSS fit for purpose													easy to use after a short learning period				
	CDSS fit for purpose						Attractive visuals and use of colour		patient-facing component of CDS		format of presentation (barrier or facilitator)			CDSS is user-friendly (ergonomic)			User-friendly design of interface	ease of navigation
	CDSS fit for purpose													pleasing visual lay-out				
	Available functionalities	CDSS use in audit, feedback and benchmarking				Feedback and audits	Functionalities to examine population and individual level data				use of the native language	Detailed documentation	provide an audit trail and support research.					
	Available functionalities					The model provides a standardized assessment and monitoring framework	Information includes historical data						CDSSs provide immediate feedback to clinicians and patients					
	Available functionalities												provide control					
Workflow	Integration into usual workflow	CDSS integration and fit with workflows				Tool quick to complete, no increase in workload		Fitting with usual workflow		fitting the model to physician workflow by streamlining patient education and orders				natural integration of the CDSS in the clinical workflow	Implementing the DA in existing structures (education session)		Adequate workflow integration	
	Integration into usual workflow					No increased burden or negative consequences												
	Time and effort to use			Time as a resource										Other professionals ease physician's increased workload with the CDSS				
	Provides structure and guidance			Investigation and referral patterns		Guidance on actions based on model outputs	Support systematic and structured processes, improving quality of care											
Perceived effect	Influences treatment outcome	perceived benefits of CDSS	improvements in accuracy of nursing problem identification .		risk refinement		Reminders improved clinician judge- ment and motivation, to provide recommended						reduce variation in the quality of care	potential to improve the quality of care		perceptions that it improved the per- formance of tasks (eg. facilitating decision-		treatment decisions were influenced by recommendations from
	Influences treatment outcome	Impact of CDSS on patient outcome or safety	high risk patients received appropriate care sooner than usual when DSS was used for										improve decisions	CDSS helps professionals to improve guideline adherence				
	Influences treatment outcome		received more oral rehydration than intravenous when DSS was implemented											CDSS facilitates decision making				
	Influences treatment outcome		more diagnosis of hypertension and diabetes															
	Influences treatment outcome		more accurate treatments' use															
	Influences treatment outcome		reduction in readmission															
	Influences treatment outcome		reduction of situations as falls or pressure injuries															
	Influences treatment outcome		significant reduction in mean systolic blood pressure and fasting blood glucose.															
	Influences communication	CDSS impact on clinician-patient relationship, communication and action		Communicating, sharing and understanding risk	communication	The model provides a framework for communication between	Facilitates clinical discussions, par- titularly in opportunities for shared						Development and facilitation of inter-professional communication	CDSS helps focus on patient education				treatment information and the relationship with survival were included to facilitate
	Influences communication	CDSS used as tool for clinician communication/education											facilitate teamwork in medical staff	CDSS eases patient-PCP communication				information provided by the technology was given to patients by physicians
	Influences communication	interdisciplinary relationship											facilitate clinical group decision-making	CDSS leads to better teamwork in primary care				a copy of the information produced by the technology was used for reference during
	Influences care process	impact of CDSS on clinician efficiently/time	reduction in time required to make a decision				Saving time and easy to implement	eCDS tools that were seen to improve efficiency				Workload assessment	help overcome problems due to inefficient coding of data	using CDSS saves time		streamlining prescribing practices		
	Influences care process							Reduced need for data entry				Improved planning						
	Increased patient and professional experience					Screening provides a sense of safety								CDSSs increase professionals satisfaction				
	Increased patient and professional experience																	
Perceived relevance	Relevant within context	contextual fit of CDSS with role/setting				Perceived utility (added value for clinical decision and/or improved quality of care)								perceived usefulness of the CDSS				technology helped physicians to understand patients' treatment preferences
	Relevant within context					ED is the best place to screen for the target condition	Useful sources of knowledge and advice during the consult							patients' [perceived usefulness of the CDSS increases professionals				
	Relevant within context																	presence of helpful information
	Relevant within context					Tool/model contributes to solving a priority health care area/problem in the region or								recommendations are relevant				
	Relevant within context					The substantial target population for implementation							ability to respond fast to unexpected situations	produced reports of quality measures through collected data increases the value from				
	Relevant within context													information provided is useful for the targeted process of care				information provided by the technology was useful to physicians.
	Information is provided timely						Patient information is immediately avail- able at point of care											
	Personalized to context					Adaptation and tailoring to suit the context			providing evidence-based and personalized recommendations for				suggesting alternative or additional treatment options,					
	Alignment with organizational goals	alignment of CDSS with organisational goals				Existing priorities and efforts in the target area												
Trustworthiness/accaptability	Reliability of information	Trust in the CDSS input and evidence-base				The model derived with sound medical and scientific basis and/or well validated								recommendations are reliable		perceptions that the digital intervention was a trusted and credible source of		confidence in the treatment plan
	Reliability of information	Trust is CDSS output and recommendations																

	**Medicolegal compliance**					The model offers medicolegal protection for patients												
	**Respect of user autonomy**				autonomy													
	**Organizational culture**	organisation support/culture				Senior Management support (± funding)			institutional support						satisfaction with the CDSS service support			Favourable organizational structures
	**Organizational culture**					Existing staff is well suited and able to support the model implementation												
	**Organizational culture**					Support and cooperation between departments (eg, clinical, IT)												
	**Attitude towards CDSS**	individual clinician role/responsibility (also barrier)													professionals would continue to use the CDSS	Clinicians' positive attitude towards preference eliciting		
	**Attitude towards CDSS**	clinicians attitudes values and preferences				Substantial interest in screening				capability, opportunity, and motivation (also a barrier)								
**sufficient training**	**Training and education**	implementation training and support for CDSS				Training and education	Receiving initial and follow-up training			educating providers about the model (also a barrier)	high educational level			Education and training	training before use is appreciated		provision of education and training	Sufficient training
	**Training and education**													medical education and training	educational materials for patients are valuable			
	**Knowledge of CDSS**			Increasing awareness			Familiarity with tool										promotion of the intervention,	
	**Knowledge of CDSS**																increased frequency of use and understanding of the intervention's capabilities,	
	**Technical skill level**											(also a barrier) comfortable with computer technology						
	**Supports knowledge**	CDSS acts to support/enhance clinician expertise				The model complements the lack of specific experience in existing staff	Perceived usefulness to expand skills							provide new learning	CDSS is a way to update PCP's knowledge			
	**Supports knowledge**					Model/tool increased vigilance and confidence in recognizing the target									CDSS helps PCPs to identify unrecognized information needs			
**Implementation strategy**	**local champion**	local champions							physician champions									
	**Implementation planning**					Dedicated implementation teams	Leadership and allocated person to oversee CDS implementation											
	**Implementation planning**	clinician engagement in planning and implementation				Early engagement of adopters	Engaging the team and maintaining staff skills		multidisciplinary teams developing the CDS			involvement of the administrator or head of nursing in the process						
	**incentives**					Competition due to implementation at other sites				providing incentives and reminders to encourage use of the model (also a barrier)								
	**Advocated by professional/regulatory body**					Tool/model advocated by a professional or regulatory body												
**Resources**	**Financial**													saving money				
	**Technical**						Providing technical support									Rooms for use of one screen together with patient		
