# Supplementary material for: The GUIDE-CDSS framework: a comprehensive framework to GUIDE and evaluate the implementation of Clinical Decision Support Systems in healthcare, based on an overview of reviews
Source: J Am Med Inform Assoc. 2026 Jun 12;33(9):1811–21. doi: 10.1093/jamia/ocag080 (PMC13580735; doi:10.1093/jamia/ocag080)
Supplement: Supplementary file 1 [file ocag080_supplementary_data.zip › Appendix_3.docx]

Appendix 3. Comparison table of Technology Adoption and Implementation frameworks to the GUIDE-CDSS.

| **GUIDE-CDSS** | **TIM** | **UTAUT** | **TAM** | **CFIR** | **TDF** |
| --- | --- | --- | --- | --- | --- |
| **Perceived relevance** | Perceived usefulness | Performance expectancy | Perceived usefulness | Inner setting | Social/professional role |
| **Workflow** | - | Effort expectancy | Perceived ease of use | Inner setting | Memory, attention, Decision process; behaviour regulation |
| **Trustworthiness** | Attitude towards using | Social influence | – | Inner setting; Outer setting | Social influences + optimism/pessimism; emotions |
| **Ease of use** | Perceived ease of use | Effort expectancy | Perceived ease of use | Characteristics of intervention | – |
| **Perceived effect** | - | – | – | Inner setting | Beliefs about consequences |
| **Training and skills** | – | – | – | Inner setting | Knowledge; skills |
| **Implementation strategy** | Infusion and organisational adoption | – | – | Implementation process; Individuals | – |
| **Resources** | Initial organisational influence | Facilitating conditions | External variables | Outer setting | Environmental context |
| - | Individual subjective factors | – | - | - | Confidence |
| **Implementation outcome** | | | | | |
| **Intention to use** | – | Behavioural intention | Behavioural intention to use | – | Intentions |
| **Actual use** | Use of technology | Use behaviour | Actual system use | – | – |
